# Rapid inactivation of the yeast Sec complex selectively blocks transport of post-translationally translocated proteins

**DOI:** 10.1016/j.jbc.2021.101171

**Published:** 2021-09-04

**Authors:** Jae Kyo Yi, Hidenobu Fujita, Elisabet C. Mandon, Reid Gilmore

**Affiliations:** Department of Biochemistry and Molecular Pharmacology, University of Massachusetts Medical School, Worcester, Massachusetts, USA

**Keywords:** endoplasmic reticulum, membrane protein, protein synthesis, protein translocation, ribosome, CPY, carboxypeptidase Y, DPAPB, dipeptidylaminopeptidase B, endo H, endoglycosidase H, ER, endoplasmic reticulum, FN3, fibronectin 3, GEV, Gal4–estrogen-binding domain–VP16 fusion protein, GPI, glycosylphosphatidylinositol, HA, hemagglutinin, ppCPY, cytoplasmic precursor form of CPY, RNS, ribosome-nascent chain, SD, synthetic minimal media containing dextrose, SR, SRP receptor, SRP, signal recognition particle, TEV, tobacco etch virus, TM, transmembrane, YPD, yeast extract–peptone–dextrose

## Abstract

The yeast endoplasmic reticulum has three distinct protein translocation channels. The heterotrimeric Sec61 and Ssh1 complexes, which bind translating ribosomes, mediate cotranslational translocation of proteins targeted to the endoplasmic reticulum by the signal recognition particle (SRP) and SRP receptor targeting pathway, whereas the heptameric Sec complex has been proposed to mediate ribosome-independent post-translational translocation of proteins with less hydrophobic signal sequences that escape recognition by the SRP. However, multiple reports have proposed that the Sec complex may function cotranslationally and be involved in translocation or integration of SRP-dependent protein translocation substrates. To provide insight into these conflicting views, we induced expression of the tobacco etch virus protease to achieve rapid inactivation of the Sec complex by protease-mediated cleavage within the cytoplasmic domain of the Sec63 protein. Protein translocation assays conducted after tobacco etch virus protease induction revealed a complete block in translocation of two well-characterized substrates of the Sec complex, carboxypeptidase Y (CPY) and Gas1p, when the protease cleavage sites were located at structural domain boundaries in Sec63. However, integration of SRP-dependent membrane protein substrates was not detectably impacted. Moreover, redirecting CPY to the cotranslational pathway by increasing the hydrophobicity of the signal sequence rendered translocation of CPY insensitive to inactivation of the Sec complex. We conclude that the Sec complex is primarily responsible for the translocation of yeast secretome proteins with marginally hydrophobic signal sequences.

Translocation of proteins across or integration of membrane proteins into the endoplasmic reticulum (ER) can occur by cotranslational or post-translational pathways in budding yeast. The most hydrophobic signal sequences including the membrane-spanning segments of integral membrane proteins are cotranslationally recognized by the signal recognition particle (SRP). The SRP and the SRP receptor (SR) function in concert to target the ribosome-nascent chain (RNC) complex to the heterotrimeric Sec61 or Ssh1 complexes. The Ssh1 heterotrimer is a nonessential cotranslational protein translocation channel (1, 2, 3). Secretome proteins with less hydrophobic signal sequences are not recognized by the SRP (4, 5) but are instead targeted to the heptameric Sec complex by cytosolic chaperones and alternative targeting factors (5, 6, 7). The Sec complex consists of the tetrameric Sec62–Sec63 complex (Sec62, Sec63, Sec71, and Sec72) plus the Sec61 heterotrimer (4, 6, 8, 9). Unlike the Sec61or Ssh1 heterotrimers, the Sec complex does not bind 80S ribosomes or interact directly with RNCs (10). Consequently, the Sec complex was initially viewed as an obligatory post-translational translocation channel (8, 9, 11, 12). Partitioning of the yeast secretome proteins between the two targeting pathways is not absolute as signal sequences of intermediate hydrophobicity can direct substrates to both pathways (4, 13).

The concept that distinct targeting pathways deliver substrates to either the heterotrimeric Sec61 complex or the heptameric Sec complex has been challenged in recent years. Ribosome-profiling assays have shown that ribosomes translating secretome mRNAs can be biotinylated *in vivo* by ER-localized Ssh1-BirA, Sec63-BirA, or Ubc6-BirA fusion proteins (14). Remarkably, ribosomes synthesizing well-characterized substrates of the yeast post-translational translocation pathway (*e.g.*, carboxypeptidase Y [CPY] and Gas1p) are enriched in the biotinylated products relative to ribosomes synthesizing mitochondrial or cytosolic proteins. One interpretation of these ribosome-profiling results is that yeast translocation reactions are primarily cotranslational regardless of the targeting components or the translocation channel (Sec61/Ssh1 heterotrimers *versus* the Sec complex) (14). Moreover, ribosome profiling of ER-bound ribosomes did not reveal a difference in distribution for ribosomes translating integral membrane proteins and yeast secretory proteins (15). These results led to the proposal that yeast SRP is responsible for ER targeting of virtually all ribosomes synthesizing secretome proteins except for the tail-anchored membrane proteins that are targeted by the guided entry of tail-anchored protein pathway (16). However, rapid inactivation of yeast SRP combined with ER-specific ribosome profiling indicated that ribosomes synthesizing SRP-independent substrates remained ER localized in SRP-deficient cells (17), whereas mRNAs encoding ER-targeted integral membrane proteins were mislocalized to the mitochondria. Thus, ER localization of polysomes that are synthesizing substrates for the Sec complex is not dependent upon the SRP–SR ribosome targeting pathway.

The cryo-EM structure of the yeast Sec complex revealed that the cytosolic fibronectin 3 (FN3) domain of Sec63 packs against L6/7 and L8/9 of Sec61p (18, 19, 20), thereby blocking the evolutionarily conserved ribosome binding site on the Sec61 heterotrimer (21, 22, 23). The interaction between the FN3 domain of Sec63p and Sec61p is a critical element in opening the lateral gate of Sec61p (19), which is in an open conformation in the absence of a translocation substrate in the Sec complex (18, 19). The tetratratricopeptide repeat domain of Sec72 can interact with a cytosolic Hsp70 protein (Ssa1p) or a ribosome-associated Hsp70 protein (Ssb1p) using nonoverlapping binding sites, indicating a direct role for Sec72p in substrate targeting to the Sec complex (24). An interaction between the tetratricopeptide repeat domain of Sec72 and the BRR2-like BRL domain of Sec63p places the Ssa1 interaction site on Sec72p roughly 60 Å from the central pore in Sec61p with an unobstructed path for the nascent chain to the transport pore (18, 19). The cryo-EM structure of a complex between a protein translocation substrate and the Sec complex revealed that TM1 of Sec62p interacts with two of the lateral gate transmembrane (TM) spans of Sec61 (TM2 and TM3), whereas TM2 of Sec62p interacts with the signal sequence (20). Moreover, signal sequence binding further expands the lateral gate opening and displaces the plug domain of Sec61p (20). Thus, the structure of the Sec complex is consistent with a post-translational, ribosome-independent, and translocation mechanism.

The J domain in the lumenal loop of Sec63p recruits the lumenal Hsp70 protein Kar2p (25) to provide a driving force for post-translational translocation (26). The C-terminal acidic domain of Sec63p interacts with the basic N terminus of Sec62p (27, 28). Short C-terminal truncations of Sec63p yield viable strains with translocation defects (*sec63Δ27* = *sec63-201* (29)), whereas larger truncations (*e.g.*, *sec63*Δ28) cause a recessive lethal phenotype (30, 31). Analysis of *sec63* truncations that extend into the FN3 domain led to the proposal that the Sec complex is required for cotranslational integration of integral membrane proteins (31). Additional support for this conclusion was provided by analysis of Sec63p depletion strains and of yeast *kar2* mutants (30, 32). More recent reports propose that the Sec complex is involved in the integration of signal anchor proteins with moderately hydrophobic TM spans (13) and for the translocation of lumenal C-terminal domains of integral membrane proteins (33, 34).

The lack of a ribosome binding site on the Sec complex is difficult to accommodate in a mechanism that proposes a general role for Sec63p in the integration of integral membrane proteins. One caveat with several previous studies that support such a model is that gene product depletion experiments do not allow rapid inactivation of the Sec complex. A second caveat is that classical *sec62*, *sec63*, or *kar2* alleles (*e.g.*, *sec62-1*, *sec63-1*) display readily detectable translocation defects even when cultured at the permissive temperature (35). Thus, the apparent involvement of the Sec complex in the integration of membrane proteins might be explained by an indirect mechanism that involves depletion of an ER protein required for cotranslational integration of proteins by the heterotrimeric protein translocation channels. To address this caveat, we developed a procedure that would allow rapid and tightly regulated inactivation of the yeast Sec complex by proteolytic cleavage of Sec63p at defined sites in the C-terminal cytosolic domain. Removal of the acidic C-terminal domain of Sec63p or removal of both the acidic domain and the FN3 domain of Sec63 caused a rapid and complete block in the translocation of CPY and Gas1p, two proteins that are translocated by the Sec complex. Proteolytic removal of these Sec63 domains did not cause a detectable defect in the integration of two type 2 membrane proteins (dipeptidylaminopeptidase B [DPAPB] or Pho8) and did not reduce translocation of secretory invertase. Our results support the hypothesis that the Sec complex does not serve as the translocation channel for the transport or integration of yeast secretome proteins that are cotranslationally targeted to the rough ER by the SRP–SR pathway.

## Results

### Regulated cleavage of Sec63p

To obtain a better understanding of the role of the Sec complex in protein translocation reactions in yeast, we developed a strategy to achieve rapid inactivation of the Sec complex by regulated cleavage within the cytoplasmically localized C-terminal domain of Sec63. As this project was initiated before the structure of the Sec complex was solved by cryo-EM (18, 19), the design of the protease insertion sites was based upon an alignment between yeast Sec63p and the DExD/H helicase Brr2, which contains two Sec63-like domains (36).

**Figure 1:**
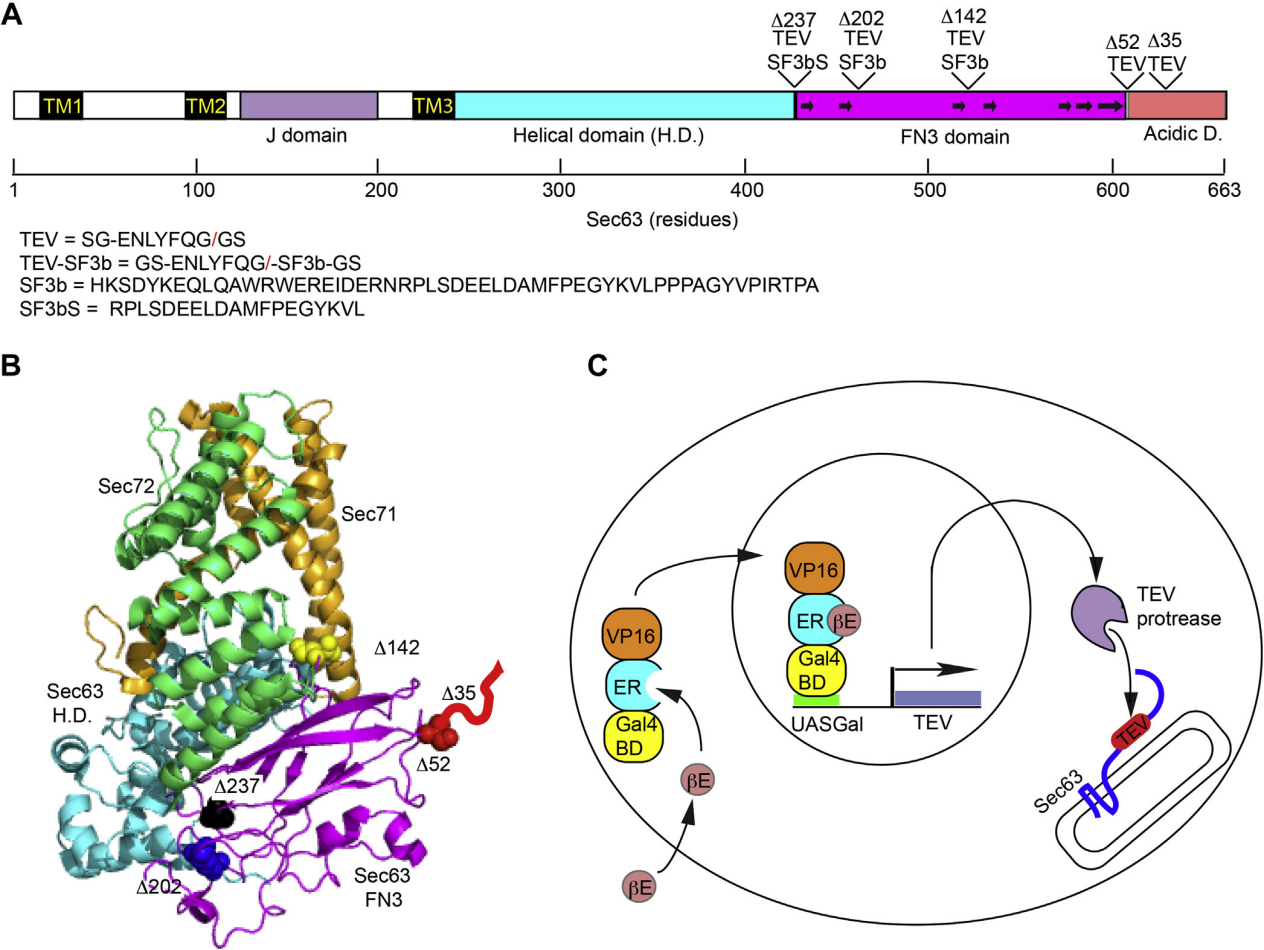
**Regulated cleavage of Sec63p in yeast cells.***A*, a diagram of yeast Sec63p showing the location of the TM spans (TM1–TM3) and structural domains. The β-strands in the FN3 domain are designated by *black arrows*. The location and sequence of the inserted TEV or TEV-SF3b protease cleavage sites are shown. *B*, the cytosolic domain of the yeast Sec62–Sec63 complex is shown to indicate the location of the TEV protease cleavage sites in the Sec63p structure. Sec71 is *orange*, Sec72 is *lime green*, Sec63 domains are color coded as in panel *A*. Sec62p and the C-terminal acidic domain of Sec63p (*red line*) were not resolved. *C*, GEV induction of TEV protease expression in yeast. Binding of β-estradiol (βE) to the estrogen receptor (ER) domain of the GEV transcriptional activator promotes nuclear entry of active GEV followed by transcription of the TEV protease mRNA. TEV protease can cleave the TEV protease sites in Sec63p. FN3, fibronectin 3; GEV, Gal4–estrogen-binding domain–VP16 fusion protein; TEV, tobacco etch virus; TM, transmembrane.

Tobacco etch virus (TEV) protease cleavage sites flanked on both sides by flexible linkers were inserted into the C-terminal acidic domain of Sec63 (Fig. 1*A*, *sec63Δ35* mutant) and at the domain boundary between the FN3 domain and the acidic domain (Fig. 1*A*, *sec63*Δ52 mutant). The mutant names (*e.g.*, *sec63Δ35*) designate the number of C-terminal residues that will be removed upon cleavage of Sec63p by the TEV protease. The acidic C-terminal domain of Sec63, which is thought to be disordered based upon analysis with the DisEMBL Web server (http://dis.embl.de), has been shown to interact with the N-terminal basic domain of Sec62p (27, 28). The Sec62–Sec63 interaction was not confirmed by the recent cryo-EM structures as both Sec62p and the C-terminal acidic domain of Sec63 were not well resolved (Fig. 1*B*).

To facilitate TEV protease recognition of cleavage sites within the structured domains of Sec63p (Fig. 1, *A* and *B*), the loop containing the TEV protease site was expanded by appending the 54-residue p14 SF3b domain adjacent to the TEV protease site (Fig. 1*A*). SF3b domains have previously been used to enhance recognition of cleavage sites by the TEV protease (37). For simplicity, we will refer to the *sec63* mutants that have the TEV-SF3b cleavage sites using the same nomenclature that specifies the number of Sec63 residues that will be removed by TEV protease cleavage (*i.e.*, *sec63*Δ142, *sec63*Δ202, and *sec63*Δ237). The SF3b domain in the *sec63*Δ237 construct was trimmed to the minimal effective size to reduce potential steric clashes with the helical or FN3 domains of Sec63p (SF3b-S in Fig. 1*A*). We used the Gal4–estrogen-binding domain–VP16 fusion protein (GEV) induction system (37) to regulate TEV protease expression (Fig. 1*C*). Unlike a galactose-inducible expression system, the GEV induction system does not cause a carbon source–dependent growth rate change, so the GEV induction system was more suitable for evaluating the impact of Sec63 cleavage upon protein translocation.

**Figure 2:**
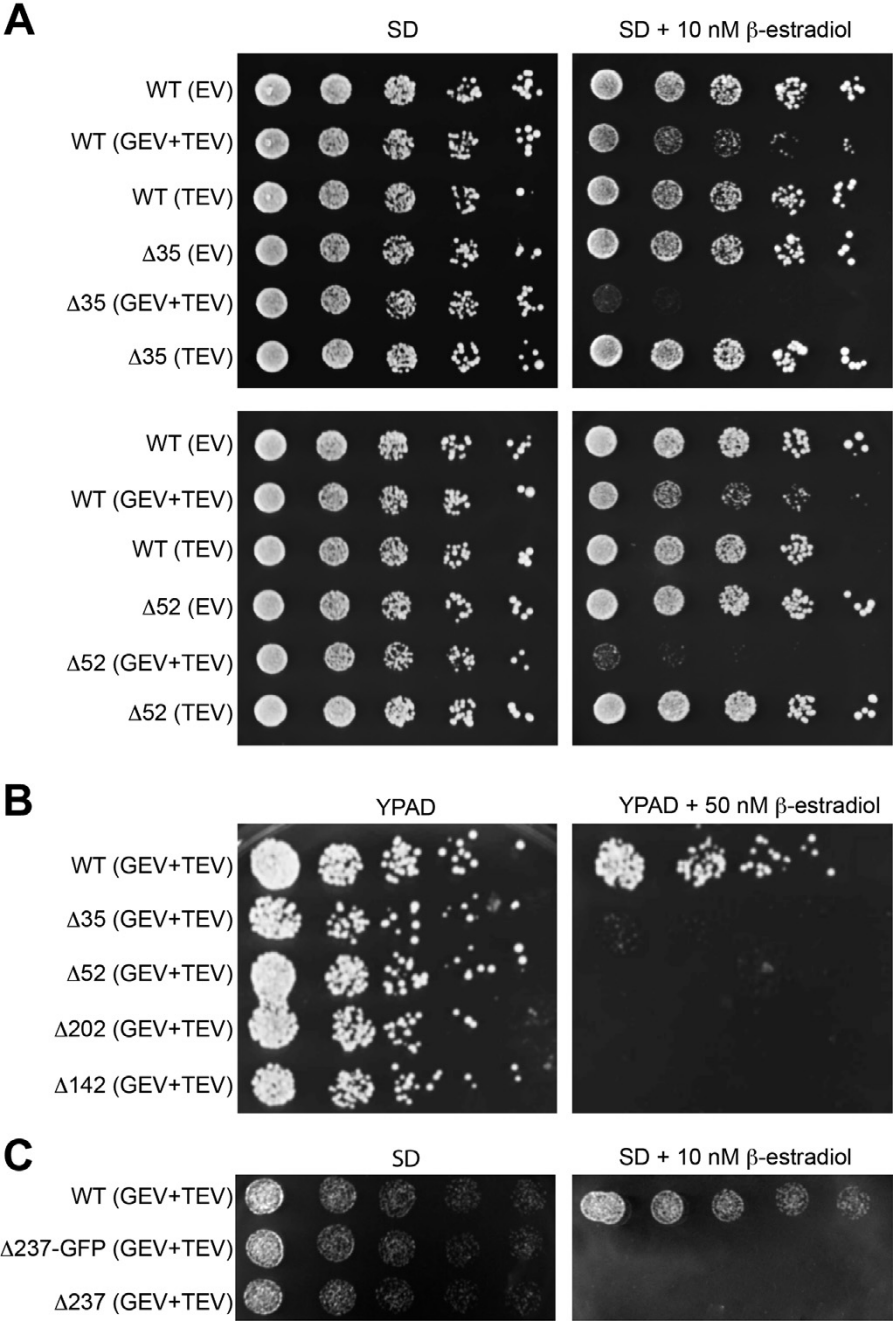
**Insertion of TEV protease cleavage sites into Sec63p causes a growth defect that is dependent upon TEV protease expression.***A*, fivefold serial dilutions of yeast strains that express wildtype (WT) or mutant alleles of Sec63p were spotted onto SD plates (±10 nM β-estradiol) to evaluate growth at 30 °C for 2 days. The yeast strains either contained both the TEV and GEV expression cassettes, just the TEV cassette, or lacked both cassettes (EV). *B*, fivefold serial dilutions of yeast strains that express WT or mutant alleles of Sec63p were spotted onto YPAD plates (±50 nM β-estradiol) to evaluate growth at 30 °C for 2 days. *C*, fivefold serial dilutions of yeast strains that express WT or mutant alleles of Sec63p were spotted onto SD plates (±10 nM β-estradiol) to evaluate growth at 30 °C for 2 days. EV, empty vector; SD, synthetic minimal media containing dextrose; TEV, tobacco etch virus; YPAD, yeast extract, peptone, and dextrose media containing adenine.

Yeast strains that contain plasmids encoding wildtype or mutant alleles of Sec63p (Δ35 and Δ52) were first tested for growth in the absence of β-estradiol to determine whether the inserted TEV protease cleavage sites in Sec63 cause a detectable growth defect for cells grown on synthetic defined media (synthetic minimal media containing dextrose [SD]). Based upon the colony dilution assay (Fig. 2*A*, *left panel*), insertion of the TEV protease recognition site at the Δ35 and Δ52 positions did not cause a growth defect. As expected, the presence of the TEV expression cassette, or both the TEV and GEV expression cassettes, did not reduce the growth of yeast strains harboring wildtype or mutant (Δ35 or Δ52) alleles of Sec63 when the media lacked β-estradiol (Fig. 2*A*, *left panel*). In the presence of β-estradiol, we observed a dramatic inhibition of growth when Sec63p contained a TEV protease cleavage site and the cells harbored both the GEV and TEV expression cassettes. A modest growth rate decrease was caused by TEV protease expression in a wildtype Sec63 strain.

Having established that β-estradiol–induced expression of TEV protease is causing a growth defect in yeast strains containing TEV protease cleavage sites in the acidic domain of Sec63p, we next tested whether the TEV-SF3b sites in the FN3 domain of Sec63p were tolerated in the absence of β-estradiol and caused a growth defect upon TEV protease expression (Fig. 2*B*). Yeast strains expressing the Sec63Δ142, Sec63Δ202, Sec63Δ237-GFP, and Sec63Δ237 proteins grew normally on plates in the absence of β-estradiol but did not form colonies when TEV protease was expressed (Fig. 2, *B* and *C*).

### Rapid cleavage of Sec63 upon TEV protease induction

**Figure 3:**
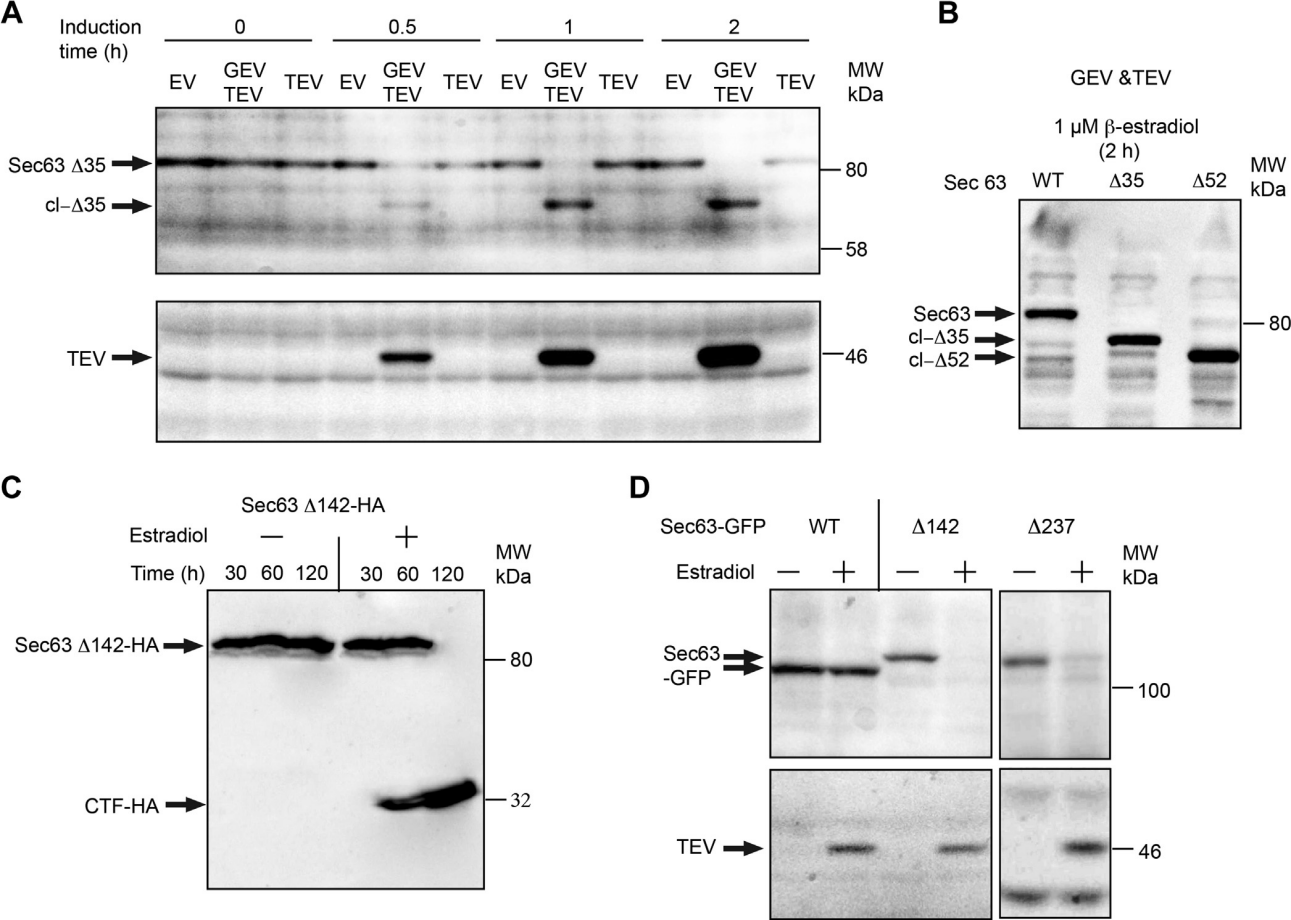
**Rapid cleavage of Sec63 in yeast strains that express TEV protease.***A*, yeast strains that express Sec63Δ35 and either lack both expression cassettes (empty vector [EV]), only contain the TEV cassette, or contain both the TEV and GEV cassettes were grown in liquid SD media for 0 to 2 h after addition of 1 μM β-estradiol. Whole-cell extracts were subjected to SDS-PAGE to separate intact Sec63p from TEV protease–derived fragments. Protein immunoblots utilized anti-Sec63 or anti-TEV protease sera. *B*, yeast strains that express wildtype or mutant Sec63p were grown for 2 h in the presence of 1 μM β-estradiol. Whole-cell extracts were subjected to SDS-PAGE, and the immunoblots were probed with anti-Sec63. *C*, a yeast strain that contained both the GEV and TEV expression cassettes and expressed Sec63Δ142-HA was grown for 0 to 120 min in the presence or the absence of 1 μM β-estradiol. Whole-cell extracts were subjected to SDS-PAGE, and the immunoblots were probed with anti-HA sera. *D*, yeast strains that contained both the TEV and GEV cassettes and expressed Sec63-GFP, Sec63Δ142-GFP, or Sec63Δ237-GFP were grown for 1 h in the presence or the absence of 1 μM β-estradiol. Whole-cell extracts were subjected to SDS-PAGE to separate intact Sec63p from TEV protease–derived fragments. Protein immunoblots were probed with anti-Sec63 or anti-TEV protease sera. GEV, Gal4–estrogen-binding domain–VP16 fusion protein; SD, synthetic minimal media containing dextrose; TEV, tobacco etch virus.

The results of the colony dilution experiments are consistent with a previous report indicating that a C-terminal truncation of more than 27 residues from Sec63p causes a lethal phenotype (31). However, as TEV protease cleavage of Sec63 could be limited to the newly synthesized protein rather than the assembled SEC complex, we used protein immunoblotting to assay the kinetics of TEV protease induction and Sec63p cleavage. TEV protease expression was detectable after 30 min of induction, and protease levels continued to increase during the 2-h time course (Fig. 3*A*). Cleavage of Sec63Δ35p was detected at the 30-min time point as indicated by the decrease in immunoblot signal for the intact protein and the appearance of a more rapidly migrating truncated product (cl-Δ35). If the TEV protease was only able to cleave newly synthesized Sec63Δ35, we would expect Sec63Δ35 cleavage to lag well behind TEV protease expression, with the intact protein persisting for 4 to 6 h as it does when Sec63p expression is under control of the repressible MET3 promoter (30, 31). The observation that cleavage was essentially complete after 1 h of induction indicated that Sec63Δ35p in the Sec complex was susceptible to cleavage by TEV protease (Fig. 3*A*). Complete cleavage of Sec63Δ52p and production of the diagnostic truncated product (cl-Δ52) was also observed after a 2-h induction (Fig. 3*B*).

While TEV protease induction resulted in cleavage of the Sec63Δ142 and Sec63Δ237 products as indicated by the disappearance of the intact protein, we were not able to detect a cleavage product with the anti-Sec63 antisera. To overcome this limitation, we constructed a hemagglutinin (HA)-tagged version of Sec63Δ142 and GFP-tagged versions of Sec63Δ142 and Sec63Δ237. Cleavage of Sec63Δ142-HA was not as rapid as cleavage of Sec63Δ35 but was complete after 2 h of TEV protease induction (Fig. 3*C*). The mobility (∼32 kDa) of the HA-tagged C-terminal fragment was consistent with cleavage at the inserted TEV-SF3b site. Cleavage of Sec63Δ142-GFP was completed by 1 h of induction, and cleavage of Sec63Δ237-GFP was complete after 2 h of induction (Fig. 3*D*). Based upon the immunoblot experiments, we conclude that the TEV protease can cleave both the newly synthesized Sec63 and pre-existing Sec63 that were assembled into the Sec complex prior to TEV protease expression.

### Defects in protein translocation across the ER

**Figure 4:**
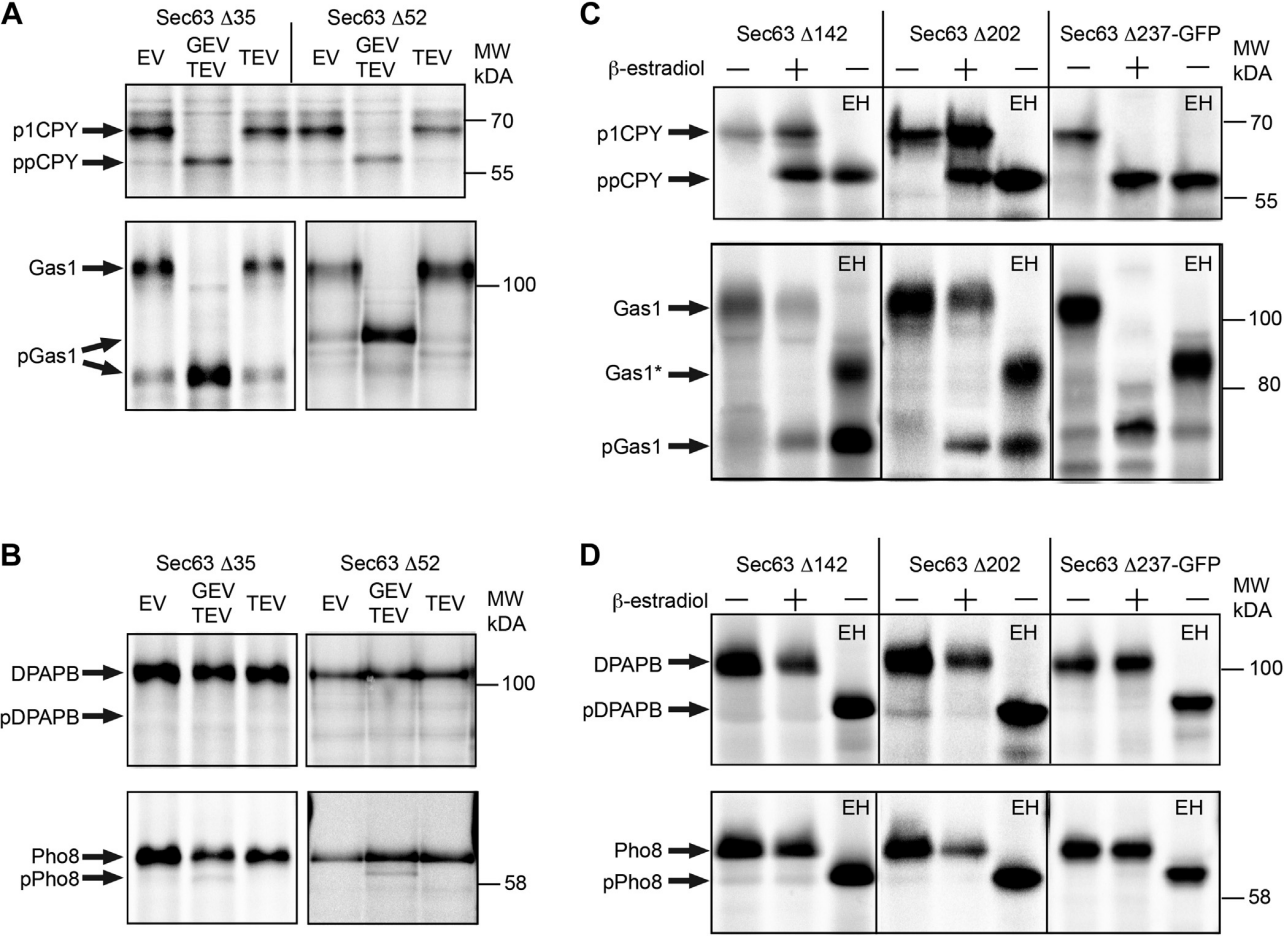
**Translocation assays of yeast strains that express TEV protease–sensitive Sec63p.** Yeast strains that express Sec63 proteins containing the indicated TEV protease cleavage sites were grown for 2 h in SD media in the absence or the presence of 1 μM β-estradiol prior to pulse labeling (7 min at 30 °C). Immunoprecipitates were resolved by SDS-PAGE to resolve the nonglycosylated precursors from translocated or integrated products. *A*, yeast strains that either contained both expression cassettes (GEV and TEV), the TEV expression cassette (TEV), or neither expression cassettes (EV) were assayed for translocation of CPY and Gas1p by pulse labeling. The two Gas1 images, which are derived from gels that were electrophoresed for different durations, have been aligned to show a common mobility for the ER form of Gas1. *B*, yeast strains that contained the indicated expression cassettes were assayed for integration of DPAPB and Pho8 by pulse labeling. *C*, yeast strains that contained both expression cassettes were assayed for translocation of CPY and Gas1p by pulse labeling. As indicated (EH), the immunoprecipitated proteins were digested with endo H to provide approximate protein mobility markers for ppCPY. Endo H digestion of Gas1 yields Gas1∗, which migrates slower than pGas1 because of the presence of a glycosylphosphatidylinositol anchor. *D*, yeast strains that contained both expression cassettes were assayed for integration of DPAPB and Pho8 by pulse labeling. As indicated (EH), the immunoprecipitated proteins were digested with endo H to provide approximate protein mobility markers for pDPAPB and pPho8. CPY, carboxypeptidase Y; DPAPB, dipeptidylaminopeptidase B; Endo H, endoglycosidase H; ER, endoplasmic reticulum; EV, empty vector; GEV, Gal4–estrogen-binding domain–VP16 fusion protein; SD, synthetic minimal media containing dextrose; TEV, tobacco etch virus.

Having established that regulated TEV protease expression causes rapid and complete cleavage of Sec63p when the TEV protease sites are located in the cytoplasmic domain, we used pulse labeling to monitor protein translocation. CPY and Gas1p are two well-characterized yeast proteins that are translocated through the Sec complex by an SRP-independent protein translocation pathway (4, 21). Translocation of CPY into the lumen of the ER is accompanied by the addition of four N-linked glycans to yield the ER form of CPY (p1CPY), which is the predominant product detected in cells after a 7-min pulse labeling procedure (3, 20, 37). Yeast cells that lack both the expression cassettes (empty vector) or only have the TEV cassette synthesize p1CPY (Fig. 4*A*, *upper panel*). Hence, the TEV protease cleavage site in these *sec63* mutants does not cause a defect in the translocation of CPY without TEV protease expression. The unglycosylated cytoplasmic precursor form of CPY (ppCPY) (38) was the predominant form of CPY upon proteolytic removal of the C-terminal acidic domain of Sec63. The glycosylphosphatidylinositol (GPI) anchored protein Gas1p receives both N-linked glycans and a C-terminal GPI anchor upon post-translational translocation into the ER lumen (4). TEV protease-mediated cleavage of Sec63 within the C-terminal acidic domain completely blocked translocation of Gas1p (Fig. 4*A*, *lower panel*).

DPAPB and Pho8p are type 2 (N_cyt_–C_lum_) integral membrane proteins that are integrated by an SRP-dependent cotranslational pathway (4, 21). As integration of both membrane proteins is accompanied by N-linked glycosylation, the ER forms migrate less rapidly than the precursor forms (Fig. 4*B*). We were unable to detect a precursor form of DPAPB and only observed traces of a Pho8 precursor in cells after induction of the TEV protease. The observation that the C-terminal acidic domain of Sec63 is not required for integration of DPAPB and Pho8 is in agreement with the previous characterization of the nonconditional *sec63-201* mutant (*e.g.*, Sec63Δ27 (4)) and the previous analysis of a Sec63-Δ28p truncation mutant (31).

Larger C-terminal Sec63 truncations (Sec63Δ52p and Sec63Δ113p) caused partial or complete blocks in DPAPB integration when expression of wildtype Sec63p was suppressed (31). Insertion of the expanded TEV-SF3b cleavage site into the FN3 domain (Sec63Δ142 and Sec63Δ202) or at the boundary between the FN3 domain and the helical domain of Sec63p (Sec63Δ237-GFP) did not cause a translocation defect in the absence of TEV protease expression (Fig. 4, *C* and *D*). Pulse labeling analysis of yeast strains that harbor TEV protease sites in the FN3 domain of Sec63 (Sec63Δ142 and Sec63Δ202 mutants) showed incomplete blocks in CPY translocation upon β-estradiol–induced expression of the TEV protease (Fig. 4*C*). An incomplete block in Gas1p translocation was also detected for both the *sec63*Δ142 and *sec63*Δ202 mutants. When the TEV protease site was located at the boundary between the helical domain and the FN3 domain of Sec63 (Sec63Δ237-GFP), TEV protease induction was accompanied by a complete block in CPY and Gas1 translocation (Fig. 4*C*). We utilized endoglycosidase H (endo H) digestion of the immunoprecipitated proteins to remove the N-glycans from p1CPY, Gas1, DPAPB, and Pho8 (Fig. 4, *C* and *D*). The endo H-digested proteins serve as approximate protein mobility markers for ppCPY, pDPAPB, and pPho8. Deglycosylated p1CPY and Gas1p lack the N-terminal signal sequences of ppCPY and pGas1. Endo H-digested glycoproteins retain one GlcNac residue for each of the original N-glycans. Endo H-digested p1CPY comigrated with the ppCPY that was detected in the immunoprecipitates from the β-estradiol-treated cells. Endo H digestion of the glycosylated ER form of Gas1p yields a protein with an intermediate gel mobility (Gas1∗) because of retention of the GPI anchor.

TEV protease cleavage within the FN3 domain or at the FN3-helical domain boundary did not cause a detectable reduction in the integration of either DPAPB or Pho8 (Fig. 4*D*), even though cleavage at these sites removes segments of Sec63p that are larger than the Sec63Δ52p and Sec63Δ113p truncation mutants that were analyzed previously (31).

**Figure 5:**
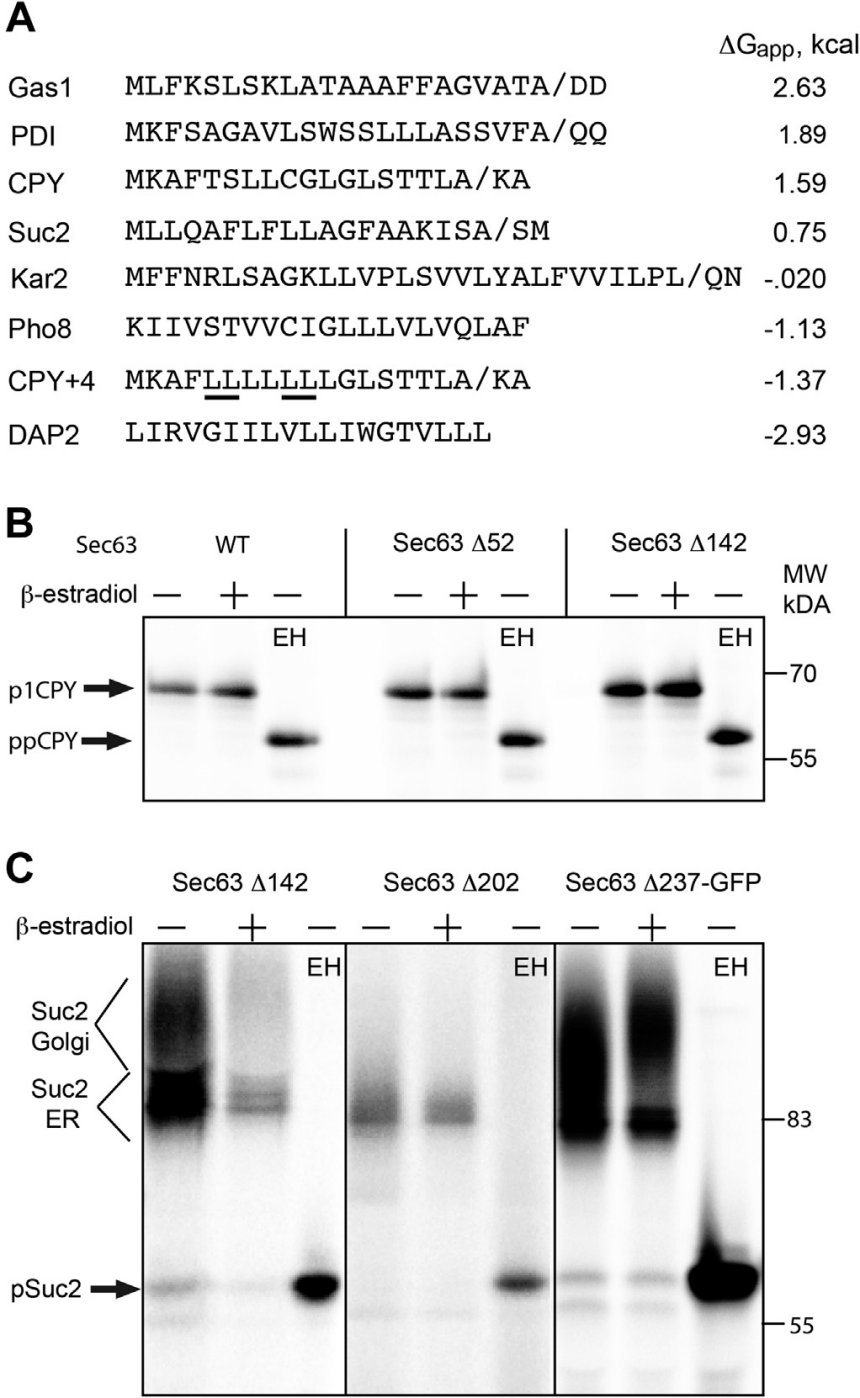
**TEV protease cleavage within the FN3 domain of Sec63p does not inhibit translocation of cotranslational pathway substrates.***A*, cleavable signal sequences or signal-anchor sequences (DAP2 and Pho8) for protein translocation substrates are ordered based upon the calculated Δ*G*_app_ value, which is a measure of hydrophobicity. The CPY+4 signal sequence was derived from the wildtype CPY signal sequence by replacing four marginally hydrophobic residues with the *underlined* leucine residues. *Diagonal lines* designate signal sequence cleavage sites. *B*, yeast strains containing both the GEV and TEV expression cassettes and express Sec63p containing the indicated TEV protease cleavage sites were grown for 2 h in SD media in the absence or the presence of 1 μM β-estradiol. Translocation of CPY+4 was assayed by pulse labeling (7 min at 30 °C). As indicated (EH), anti-T7 immunoprecipitates were digested with Endo H prior to electrophoresis to provide approximate protein mobility markers for the nontranslocated precursor (ppCPY+4-T7). *C*, yeast strains containing both the GEV and TEV expression cassettes and express *sec63* alleles containing the indicated TEV protease cleavage sites were grown for 2 h in SD media in the absence or the presence of 1 μM β-estradiol. Translocation of Suc2-HA was assayed by pulse labeling (7 min at 30 °C). As indicated (EH), anti-HA immunoprecipitates were digested with endo H prior to electrophoresis to provide an approximate protein mobility marker for the nontranslocated precursor (pSuc2). The ER and Golgi-localized forms of Suc2 are indicated by the *labeled brackets*. CPY, carboxypeptidase Y; Endo H, endoglycosidase H; ER, endoplasmic reticulum; FN3, fibronectin 3; GEV, Gal4–estrogen-binding domain–VP16 fusion protein; HA, hemagglutinin; SD, synthetic minimal media containing dextrose; TEV, tobacco etch virus.

Partitioning of yeast secretome proteins between the SRP–SR–dependent cotranslational translocation pathway and the Sec complex-dependent post-translational pathway is based upon the hydrophobicity of the signal sequence (4). Based upon pulse labeling experiments conducted using the *sec63-201* (*e.g.*, *sec63Δ27*), *sec62-101*, *sec65-19* (SRP19), and *sec61-101* mutants, several yeast proteins (CPY, Gas1, and PDI) with less hydrophobic signal sequences were shown to be translocated by an SRP-independent pathway that utilizes the Sec complex (4). DPAPB (DAP2) and Pho8p were translocated by a cotranslational pathway, whereas certain proteins like Kar2p utilized both targeting pathways. When evaluated using a biological hydrophobicity scale (39), the CPY, PDI, and Gas1 signal sequences are considerably less hydrophobic than the TM spans of Pho8 or Dap2, whereas the Kar2p and Suc2p signal sequences have an intermediate hydrophobicity (Fig. 5*A*). Replacement of four marginally hydrophobic residues in the CPY signal sequence with leucine residues yields the CPY+4 signal sequence, which is similar in hydrophobicity to the TM span of Pho8p. The CPY+4 signal sequence has been shown to target RNCs synthesizing CPY to the ER by the SRP–SR targeting pathway (38).

The CPY+4-T7 reporter was expressed in the strains that have cleavable forms of Sec63p to determine whether altering the targeting pathway for CPY translocation eliminates the impact of the Sec63 cleavage. In contrast to wildtype CPY, the complete removal of the C-terminal acidic domain (*sec63Δ52*) or TEV protease–mediated cleavage within the FN3 domain (*sec63*Δ142) did not cause the accumulation of the CPY precursor when the CPY+4 signal sequence was present (Fig. 5*B*). Endo H digestion confirmed that the p1CPY-T7 product contained N-linked glycans.

The signal sequence for invertase (Suc2p) has an intermediate hydrophobicity (Fig. 5*A*) raising the possibility that Suc2 might utilize both targeting pathways. However, previous analysis of invertase translocation utilizing *sec62-1*, *sec63-1*, *sec71Δ*, and *sec72Δ* mutants has yielded disparate observations about the importance of the Sec complex in invertase translocation (35, 40, 41, 42) with partial translocation defects displayed by the *sec62-1* and *sec71Δ* mutants at 37 °C. However, assays using ubiquitin-based translocation reporters indicate that invertase is translocated by an SRP-dependent cotranslational pathway (43, 44). Here, we tested whether rapid inactivation of Sec63p by TEV protease–mediated cleavage within the FN3 domain or at the FN3-helical domain boundary has an impact upon invertase translocation (Fig. 5*C*). On average, ten of the 13 glycosylation acceptor sites in invertase are modified upon translocation of Suc2p into the ER lumen (45) giving rise to the heterogeneous cluster of bands labeled as the ER form of Suc2 (Fig. 5*C*). Extensive mannosylation of the N-linked oligosaccharides of invertase in the Golgi (45) yields a diffuse high–molecular weight smear (Suc2 Golgi). Endo H digestion confirmed that the immunoprecipitated products identified as Suc2 Golgi and Suc2 ER contain N-linked glycans. Deglycosylated Suc2, which lacks the N-terminal signal sequence, provides an approximate mobility marker for pSuc2. We did not observe accumulation of the nontranslocated precursor (pSuc2), in the absence or the presence of TEV protease expression, indicating that the complete removal of the FN3 domain and C-terminal acidic cluster of Sec63p did not interfere with translocation of invertase (Fig. 5*C*).

### Cleavage of Sec63-GFP constructs

**Figure 6:**
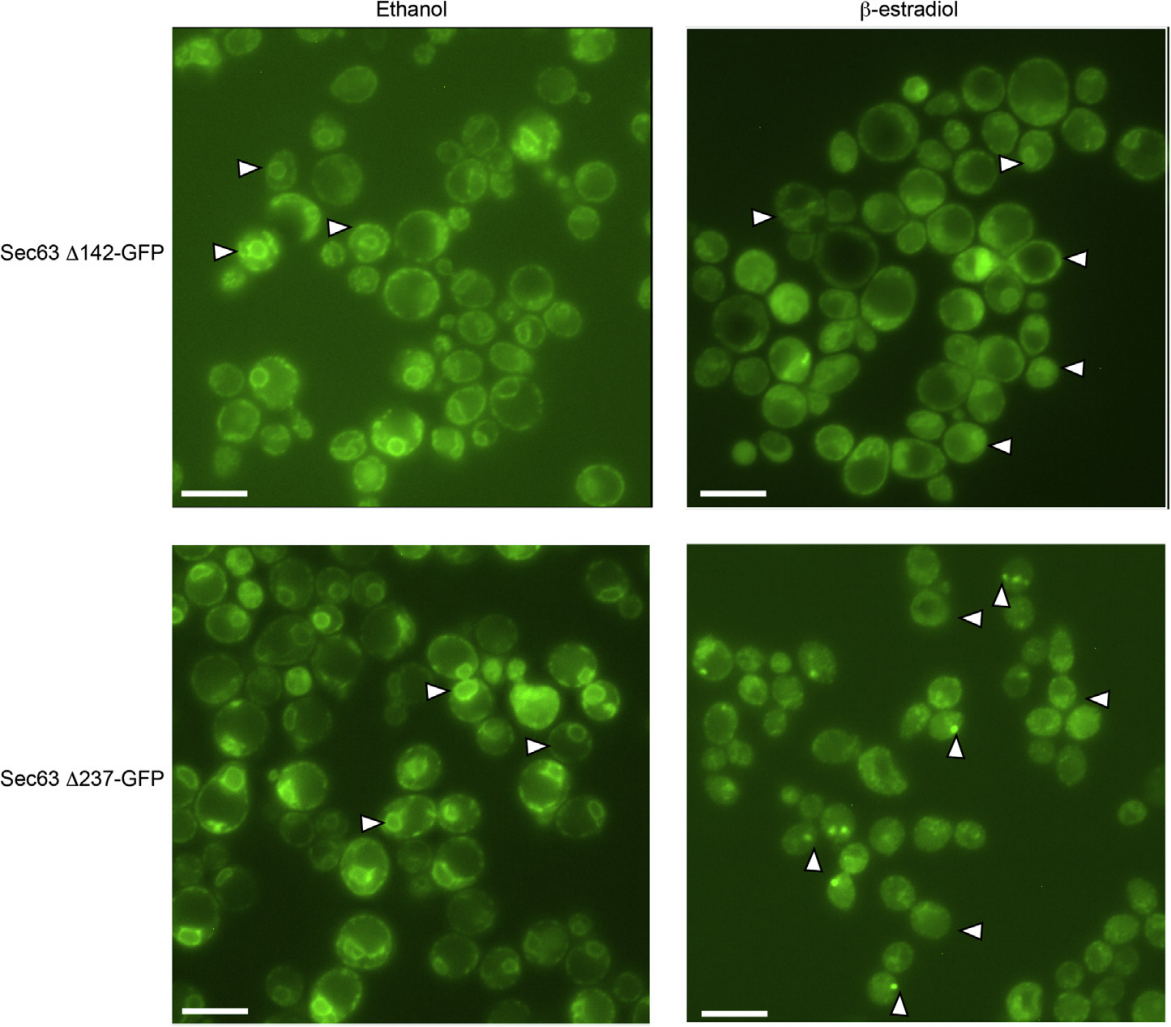
**Immunofluorescence localization of GFP-tagged Sec63.** Yeast strains that contain both the GEV and TEV expression cassettes and express GFP-tagged Sec63p containing the indicated TEV protease cleavage sites were grown for 2 h in SD media in the absence or the presence of 10 μM β-estradiol. The cells were prepared for immunofluorescence microscopy as indicated in the Experimental procedures section. The scale bar corresponds to 5 μM. *Rightward pointing arrowheads* indicate GFP localization to the nuclear envelope or the cortical ER. *Leftward pointing arrowheads* are examples of diffuse GFP immunofluorescence in cells treated with β-estradiol. *Upward pointing arrowheads* designate bright puncta in *sec63*Δ237-GFP cells that were treated with β-estradiol. ER, endoplasmic reticulum; GEV, Gal4–estrogen-binding domain–VP16 fusion protein; SD, synthetic minimal media containing dextrose; TEV, tobacco etch virus.

The observation that TEV protease–mediated cleavage within the FN3 domain of Sec63 has less impact upon translocation of CPY and Gas1p than by cleavage at the FN3-helical domain junction prompted us to determine whether C-terminal segments of the Sec63Δ142 and the Sec63Δ237 mutants were dissociating from the ER-localized Sec complex. To address this question, we obtained immunofluorescence microscopy images of yeast cells expressing either the Sec63Δ142-GFP or Sec63Δ237-GFP constructs. In the absence of β-estradiol treatment, the immunofluorescence from the GFP-tagged Sec63 constructs showed a typical yeast ER staining pattern with Sec63-GFP localized to both the nuclear envelope and the cortical ER (Fig. 6, *left-hand images*). When the Sec63Δ142-GFP–expressing cells were treated with β-estradiol, we observed an increase in the staining of the cytosolic compartment in most cells and a decrease in staining of the nuclear envelope and cortical ER. When the Sec63Δ237-GFP–expressing cells were treated with β-estradiol, we observed one or two bright puncta in many cells and a more obvious reduction in localization of GFP to the nuclear envelope and the cortical ER (Fig. 6, *right-hand images*). We speculate that the puncta might correspond to aggregated clusters of the GFP-tagged C-terminal domains of Sec63. The partial retention of an ER staining pattern in the *sec63*Δ142-GFP mutant suggests that the C-terminal fragment of Sec63 is not efficiently dissociating from the Sec complex when the cleavage occurs within the folded FN3 domain. The incomplete dissociation of the C-terminal fragment of Sec63 likely explains the incomplete block in CPY and Gas1 translocation that was observed for the *sec63*Δ142-GFP and *sec63*Δ202-GFP mutants (Fig. 4).

## Discussion

The role of the yeast Sec complex in the translocation of proteins across or integration of proteins into the ER was initially thought to be limited to secretome proteins with less hydrophobic signal sequences. Poor recognition of these marginally hydrophobic signal sequences by the yeast SRP directs proteins like CPY or Gas1p into the Sec complex–dependent post-translational translocation pathway. However, a growing number of publications have challenged the view that distinct secretome protein targeting pathways deliver substrates to cotranslational or post-translational translocation channels (14, 15, 16). It has also been reported that the Sec complex is required for the integration of membrane proteins that are targeted to the ER by the SRP–SR pathway. Here, we have described a procedure to achieve rapid and regulated inactivation of the Sec63 protein in yeast strains that lack a pre-existing growth or protein translocation defect.

The C-terminal acidic domain of Sec63p has been shown to interact with the N-terminal domain of Sec62p (27, 28, 29) on a basic patch that is part of the β-barrel domain of Sec62p (20). Native coimmunoprecipitation of Sec62p with the Sec complex is reduced in a yeast strain that expresses the *sec63*Δ27 mutant (*i.e.*, *sec63-201* (29)). The reduced stability of the heptameric Sec complex is thought to explain the post-translational translocation defect displayed by *sec63-201* strain and the lethal phenotypes that are caused by larger C-terminal truncations within the acidic domain of Sec63p. Rapid inactivation of Sec63p by TEV protease–mediated cleavage of the Sec63Δ35p and Sec63Δ52p caused a severe growth defect and a complete block in translocation of CPY and Gas1p but did not impact the integration of DPAPB, Pho8, or interfere with translocation of CPY when targeted to the ER by the CPY+4 signal sequence. Consistent with previous reports (4, 31), the Sec62–Sec63 interaction that is mediated by the C-terminal acidic domain of Sec63p is essential for the translocation of substrates that do not use the SRP–SR targeting pathway.

Although insertion of TEV protease cleavage sites into loops that are located within the FN3 domain of Sec63p yielded *sec63* alleles (*sec63*Δ142 and *sec63*Δ202) that are inviable when TEV protease expression is induced, these insertion sites did not cause rapid and complete inactivation of Sec complex despite the relatively rapid cleavage of Sec63p at the inserted TEV protease site. Based upon immunofluorescence microscopy of Sec63Δ142-GFP, we conclude that the TEV protease–generated C-terminal fragment of Sec63Δ142 does not dissociate from the preassembled Sec complex as indicated by partial retention of ER localization. Prolonged growth of the *sec63*Δ142 strain in the presence of estradiol on solid media does arrest growth presumably because of the eventual turnover of Sec complexes that contain the proteolytically cleaved form of Sec63. The FN3 domain of Sec63 contains several insertions between the β-strands relative to the homologous Sec63-like domain in BRR2. One of these insertion segments has been named the lasso (19) and contains a residue (E482) that makes a critical contact with L6/7 of Sec61. The TEV protease cleavage site in Sec63Δ202 is located at the junction between the second β-strand in the FN3 domain and the lasso. The incomplete block in CPY and Gas1p translocation that occurs upon cleavage of Sec63*Δ202* indicates that cleavage of Sec63 at this junction does not inactivate the preassembled Sec complex.

In contrast to the Sec63Δ142 and Sec63Δ202 proteins, TEV protease–mediated cleavage of Sec63 at the boundary between the helical and FN3 domains (Sec63Δ237-GFP) caused a complete block in translocation of CPY and Gas1p. The immunofluorescence microscopy analysis of *sec*63Δ237-GFP strain indicated that the cleaved C-terminal fragment of Sec63p was no longer associated with the nuclear envelope or the cortical ER but was instead located in the cytosol showing cytoplasmic foci. While previous publications have reported that truncation of Sec63p within the FN3 domain prevented the integration of DPAPB, we observed that TEV protease–mediated inactivation of Sec63Δ237-GFP does not cause a detectable inhibition of membrane protein integration (DPAPB and Pho8) or translocation of invertase. Although we have not tested whether CPY+4 translocation is impacted by cleavage of Sec63Δ237-GFP, CPY+4 translocation was not detectably reduced in the s*ec63*Δ52 and *sec63*Δ142 mutants. As these unaffected substrates are all targeted to the rough ER by the SRP–SR targeting pathway, this indicates that the Sec complex is not coupled to the SRP–SR targeting pathway regardless of whether the protein is a membrane protein (DPAPB and Pho8) or a secreted protein (Suc2 and CPY+4).

While it is well established that DPAPB integration is not defective in the *sec63-201* mutant (4), DPAPB mutants that have less hydrophobic TM spans behave as dual-pathway substrates as indicated by reduced integration in both the *sec62-1* and *sec65-1* mutants (13). It should be noted that the signal sequence of Suc2 is less hydrophobic than the dual-pathway substrate Kar2p (Fig. 5*A*), and several of the artificial leucine/alanine TM spans (*e.g.*, 6L/11A) that were tested in the context of DPAPB (13). If the critical determinant for Sec63-dependent translocation is partitioning of substrates between SRP-dependent and SRP-independent pathways, we predict that translocation of authentic dual-pathway substrates like Kar2p would be partially defective upon TEV protease–mediated cleavage of Sec63Δ35, Sec63Δ52, and Sec63Δ237-GFP. Type 1 integral membrane proteins that are targeted by cleavable N-terminal signal sequences are likely dual-pathway substrates because of the lower potential hydrophobicity of the signal sequence. Here, we have focused our analysis on translocation substrates that partition strongly into either the cotranslational or the post-translational translocation pathways.

Our working hypothesis was that the Sec complex–dependent integration of DPAPB that has been reported previously was explained either by a pre-existing defect in Sec complex–dependent post-translational translocation or by the slow inactivation of the Sec complex that occurs in gene product depletion experiments. In either case, accumulation of untranslocated secretome precursors in the cytosol or depletion of resident ER proteins including chaperones could conceivably inhibit cotranslational translocation through the Sec61 or Ssh1 heterotrimers by an indirect mechanism. The regulated and rapid inactivation of Sec63p that we have achieved here avoids both potential problems. We have not tested whether TEV-mediated cleavage of Sec63 in the *sec63*Δ35, *sec63*Δ52, or *sec63*Δ237-GFP strains will eventually cause a cotranslational translocation defect after a prolonged incubation. The GEV expression system does not appear to be optimal for prolonged incubation in liquid media because of the potential accumulation of cells that display reduced expression of the TEV protease.

Cotranslational integration of membrane proteins by the Sec61 or Ssh1 heterotrimers minimizes the exposure of hydrophobic TM spans to the cytosol. It is unclear how an RNC that is targeted to the ER by the SRP–SR pathway could be redirected to the Sec complex while avoiding cytosolic exposure of the TM span. The lack of a ribosome binding site on the Sec complex would necessitate that the RNC remains indirectly tethered to the Sec complex solely. It is unclear how indirect tethering would prevent cytosolic exposure of the TM span. This is a particularly critical problem for multispanning membrane proteins where adjacent TM spans could promote cytosolic aggregate formation. Our results do not support a role for the Sec complex in the integration of membrane proteins that are SRP pathway substrates but instead confirm that the C-terminal domain of Sec63p is critical for translocation of well-characterized substrates of the post-translational translocation pathway.

## Experimental procedures

### Strain and plasmid construction

Standard yeast media (yeast extract–peptone–dextrose [YPD] and SD), supplemented as noted, were used for growth and strain selection (46). Yeast cells were grown at 30 °C in YPD media or in SD media with continuous shaking at 250 rpm if not stated otherwise. All strains used in this study and their genotypes are presented in Table S1, and all oligonucleotides used in plasmid and strain constructions are listed in Table S2.

### β-estradiol regulation of TEV protease expression

The estrogen-inducing gene expression system was created as described (47). Briefly, GEV was integrated into the genomic *leu2*Δ*1* allele by transforming the plasmid pAGL (47) into JKS77 (*MATa ura3-52 lys2-801am ade2-101oc trp1*Δ*1 his-A200 leu2*Δ*1 sec63*Δ::*KANXM SEC63-URA-pRS315*). pAGL was linearized with the restriction enzyme EcoRV and integrated into cells followed by nourseothricin *N*-acetyl transferase selection to generate JKS74 (*leu2*Δ*1*::pAGL-GEV-NatMX). The chromosomal *GAL1* open reading frame was deleted and replaced with a TEV protease expression construct that contains P_GAL1_-TEV-*CYC1*. The TEV construct was PCR amplified by PCR from a genomic DNA extract of DBY12055 (37) using the primers upstream-GAL1 and downstream-GAL1 and transformed into strain JKS74, selecting for hygromycin B resistance, to generate JKS94 (*leu2*Δ*1*::pAGL-GEV-NatMX *gal1*Δ::TEV-HphMX). To make strains harboring the TEV protease recognition site at different locations in Sec63p, JKS94 was transformed with mutagenized pRS316-LEU2-SEC63 plasmids, that had been generated by Q5 Site-Directed Mutagenesis Kit (New England Biolabs) to insert the TEV protease recognition site with or without the SF3b domain (37) into the *SEC63* gene using the indicated primer sets (Table S2). The initial Sec63-Δ237-SF3b construct that was obtained using primers Sec63-Δ237(GS-TEVs-GS-SF3b)-F and Sec63-Δ237(GS-TEVs-GS-SF3b)-R served as a PCR template for two rounds of PCR-mediated deletion mutagenesis using primers Sec63-Δ237-short-1F, Sec63-Δ237-short-IR, Sec63-Δ237-short 2F, and Sec63-Δ237-short 2R. Following ligation, the resulting construct (*sec63*Δ237 SF3b-S) only contains the core residues in the SF3b domain that enhance TEV protease recognition of the cleavage site. The transformants were grown on SD plates supplemented with 5-fluoroorotic acid to allow plasmid shuffling. The new *sec63* mutants were confirmed by replica plating. All constructs were confirmed by sequencing.

### C-terminal tagging of Sec63

A triple HA epitope tag was appended to the C terminus of the *sec63*Δ142 mutant by recombinant PCR using the Q5 Site-Directed Mutagenesis Kit (New England Biolabs), the *sec63*Δ142 coding sequence in pRS315 and the Sec63-3XHA-F and Sec63-3XHA-R PCR primers. The PCR products were ligated and transformed into JKS94 to generate JKS127 (3X-HA). Tagging of Sec63p with GFP was done as described (48). Briefly, the dsDNA form of the GFP gene was constructed (Integrated DNA Technology) and PCR amplified using primers, ymUKG-F-Phospho and ymUKG-R-Phospho. After the PCR products were purified, the ligation reaction was performed in a 20-μl volume using 150 ng of the purified PCR product and 50 ng of pRS315-*sec63* plasmid that was linearized by PCR amplification using 26′-RE-R and Sec63-RE-blunt-F followed by DpnI treatment.

The TRP1-marked expression plasmids for DPAPB-HA (pEM778) and Pho8-T7 (pEM953) were derived from the URA3-marked expression plasmids pDN317 and pEM807, which were described previously (21, 38). The GPD promoter and protein-coding sequences from pDN317 and pEM807 were subcloned into pRS414. The plasmid pEM952 contains the GPD promoter, Gas1p coding sequence followed by a C-terminal FLAG tag in the pRS414 vector. The plasmid pRM988 contains the GDP promoter, Suc2p coding sequence followed by an SGSG flexible linker, and a C-terminal HA tag.

### Cell labeling and immunoprecipitation

Yeast strains expressing *sec63* alleles were transformed with one of the following plasmids (pEM778, pEM952, pEM953, pEM519, or pEM988) for expression of DPAPB-HA, Gas1-FLAG, Pho8-T7, ppCPY+4-T7, or Suc2-HA, respectively. For wildtype CPY, the endogenous protein was used for cell labeling and immunoprecipitation. Cells were grown at 30 °C to an absorbance of 0.4 at 600 nm and were either untreated (ethanol control) or were treated with 1 μM β-estradiol to induce TEV protease expression. After 2 h, the cells at a density of 0.6 to 0.7 at an absorbance at 600 nm were collected by centrifugation, resuspended in fresh SD media (±1 μM β-estradiol) at a density of 4.0 at an absorbance at 600 nm, and allowed to recover at 30 °C for 30 min. Four units of cells at an absorbance at 600 nm were collected by centrifugation and resuspended in 5 ml of SD media containing 0.1% dextrose and incubated for 30 min at 30 °C. Cells were then pulse labeled for 7 min with 100 μCi/OD Tran-^35^S-label. Radiolabeling experiments were terminated by dilution of the culture with an equal volume of ice-cold 20 mM NaN_3_, followed by snap freezing in liquid nitrogen. Rapid lysis of cells with glass beads and immunoprecipitation of yeast proteins was performed as described previously (49). CPY and DPAPB were immunoprecipitated using protein-specific antisera validated in previous studies (21, 50). Gas1-FLAG, Suc2-HA, Pho-T7, and CPY+4-T7 were immunoprecipitated using antisera specific for the FLAG (Sigma; F3165 anti-FLAG M2), HA (Roche; 11867423001), and T7 (Covance) epitope tags. Immunoprecipitated proteins were resolved by SDS-PAGE. Dry gels were exposed to a phosphor screen, scanned in a Typhoon FLA-9000 Fluorescent Image Analyzer (GE Healthcare), and quantified using ImageQuant software (GE Healthcare). Molecular masses of protein products on SDS-PAGE gels were estimated relative to prestained molecular mass markers (New England Biolabs).

### Spot assay

To evaluate growth rates, yeast strains were cultured in SD media at 30 °C to midlog phase. After dilution of cells to 0.1 at an absorbance at 600 nm, 5 μl aliquots of fivefold serial dilutions were spotted onto YPD or SD plates that were supplemented with β-estradiol (10 nM or 50 nM, as indicated). The plates were incubated at 30 °C for 2 days. Photographs of the plates were taken using an Amersham Imager 600 imager (GE Healthcare).

### Immunofluorescence microscopy

The intracellular localization of the Sec63p-GFP fusion protein was determined by immunofluorescence microscopy. *Saccharomyces cerevisiae* cells expressing Sec63Δ142-GFP or Sec63Δ237-GFP were grown at 30 °C to 0.4 at an absorbance at 600 nm and untreated (vehicle control) or were treated with 1 μM β-estradiol for 2 h to induce TEV protease expression. The cell cultures were then centrifuged for 5 min at 3000 rpm, washed 2× with sterile distilled water, and resuspended in SD medium (±1 μM β-estradiol) before mounting on slides. Still images of GFP-labeled cells were taken at 63× magnification with a Zeiss Z1 Inverted Fluorescence Microscope using a FITC/GFP filter. In all cases, fluorescent images were focused at the equatorial plane of the cells, and exposure was set to 500 ms.

## Data availability

All data are indicated in the article.

## Supporting information

This article contains supporting information (37, 38).

## Conflict of interest

The authors declare that they have no conflicts of interest with the contents of this article.
