# Supplementary material for: Rapid inactivation of the yeast Sec complex selectively blocks transport of post-translationally translocated proteins
Source: J Biol Chem. 2021 Sep 4;297(4):101171. doi: 10.1016/j.jbc.2021.101171 (PMC8503631; doi:10.1016/j.jbc.2021.101171)
Supplement: Supplemental Table S1 [file mmc1.docx]

**Table S1**. List of Yeast Strains

| **Strain** | | **Genotype** | **Source** |
| --- | --- | --- | --- |
| DBY12055 | | MATa, NDeg-MET4-13Myc-KanMX, gal4Δ::LEU2, gal1Δ::TEV-HphMX4, leu2Δ0::PACT1- GEV-NatMX, HAP1+ | McIsaac et al.  (2011) |
| EMy159 | | MATa ura3-52 lys2-801am ade2-101oc trp1Δ1 his-A200 leu2Δ1 | Trueman et al.  2012 |
| JKS77 | | MATa ura3-52 lys2-801am ade2-101oc trp1Δ1 his-A200 leu2Δ1 sec63Δ::KANXM SEC63wt-pEM837(Ura) | This study |
| JKS74 | | MATa ura3-52 lys2-801am ade2-101oc trp1Δ1 his-A200 sec63Δ::KANXM SEC63wt-pEM837(Ura) gal1Δ::TEV-HphMX4 leu2Δ0::PACT1-GEV-NatMX | This study |
| JKS94 | MATa ura3-52 lys2-801am ade2-101oc trp1Δ1 his-A200 sec63Δ::KANXM SEC63wt-pEM837(Ura) gal1Δ::TEV-HphMX4 leu2Δ0::PACT1-GEV-NatMX | | This study |
| JKS58 | | MATa ura3-52 lys2-801am ade2-101oc trp1Δ1 his-A200 sec63Δ::KANXM SEC63wt-pEM837(Ura) SEC63-Δ35-pRS315(LEU) gal1Δ::TEV-HphMX4 leu2Δ0::PACT1-GEV-NatMX pEM778(DPAPB) | This study |
| JKS61 | | MATa ura3-52 lys2-801am ade2-101oc trp1Δ1 his-A200 sec63Δ::KANXM SEC63wt-pEM837(Ura) SEC63-Δ35-pRS315(LEU) gal1Δ::TEV-HphMX4 leu2Δ0::PACT1-GEV-NatMX pEM952(GAS1) | This study |
| JKS64 | | MATa ura3-52 lys2-801am ade2-101oc trp1Δ1 his-A200 sec63Δ::KANXM SEC63wt-pEM837(Ura) SEC63-Δ35-pRS315(LEU) gal1Δ::TEV-HphMX4 leu2Δ0::PACT1-GEV-NatMX pEM953(PHO8) | This study |
| JKS67 | | MATa ura3-52 lys2-801am ade2-101oc trp1Δ1 his-A200 sec63Δ::KANXM SEC63wt-pEM837(Ura) SEC63-Δ35-pRS315(LEU) gal1Δ::TEV-HphMX4 leu2Δ0::PACT1-GEV-NatMX pEM988(SUC2) | This study |
| JKS69 | | MATa ura3-52 lys2-801am ade2-101oc trp1Δ1 his-A200 sec63Δ::KANXM SEC63wt-pEM837(Ura) SEC63-Δ35-pRS315(LEU) gal1Δ::TEV-HphMX4 leu2Δ0::PACT1-GEV-NatMX | This study |
| JKS46 | | MATa ura3-52 lys2-801am ade2-101oc trp1Δ1 his-A200 sec63Δ::KANXM SEC63wt-pEM837(Ura) SEC63-Δ52-pRS315(LEU) gal1Δ::TEV-HphMX4 leu2Δ0::PACT1-GEV-NatMX pEM778(DPAPB) | This study |
| JKS49 | | MATa ura3-52 lys2-801am ade2-101oc trp1Δ1 his-A200 sec63Δ::KANXM SEC63wt-pEM837(Ura) SEC63-Δ52-pRS315(LEU) gal1Δ::TEV-HphMX4 leu2Δ0::PACT1-GEV-NatMX pEM952(GAS1) | This study |
| JKS52 | | MATa ura3-52 lys2-801am ade2-101oc trp1Δ1 his-A200 sec63Δ::KANXM SEC63wt-pEM837(Ura) SEC63-Δ52-pRS315(LEU) gal1Δ::TEV-HphMX4 leu2Δ0::PACT1-GEV-NatMX pEM953(PHO8) | This study |
| JKS55 | | MATa ura3-52 lys2-801am ade2-101oc trp1Δ1 his-A200 sec63Δ::KANXM SEC63wt-pEM837(Ura) SEC63-Δ52-pRS315(LEU) gal1Δ::TEV-HphMX4 leu2Δ0::PACT1-GEV-NatMX pEM988(SUC2) | This study |
| JKS70 | | MATa ura3-52 lys2-801am ade2-101oc trp1Δ1 his-A200 sec63Δ::KANXM SEC63wt-pEM837(Ura) SEC63-Δ52-pRS315(LEU) gal1Δ::TEV-HphMX4 leu2Δ0::PACT1-GEV-NatMX | This study |
| JKS99 | | MATa ura3-52 lys2-801am ade2-101oc trp1Δ1 his-A200 sec63Δ::KANXM SEC63wt-pEM837(Ura) SEC63-Δ142-pRS315(LEU) gal1Δ::TEV-HphMX4 leu2Δ0::PACT1-GEV-NatMX pEM778(DPAPB) | This study |
| JKS100 | | MATa ura3-52 lys2-801am ade2-101oc trp1Δ1 his-A200 sec63Δ::KANXM SEC63wt-pEM837(Ura) SEC63-Δ142-pRS315(LEU) gal1Δ::TEV-HphMX4 leu2Δ0::PACT1-GEV-NatMX pEM952(GAS1) | This study |
| JKS101 | | MATa ura3-52 lys2-801am ade2-101oc trp1Δ1 his-A200 sec63Δ::KANXM SEC63wt-pEM837(Ura) SEC63-Δ142-pRS315(LEU) gal1Δ::TEV-HphMX4 leu2Δ0::PACT1-GEV-NatMX pEM953(PHO8) | This study |
| JKS102 | | MATa ura3-52 lys2-801am ade2-101oc trp1Δ1 his-A200 sec63Δ::KANXM SEC63wt-pEM837(Ura) SEC63-Δ142-pRS315(LEU) gal1Δ::TEV-HphMX4 leu2Δ0::PACT1-GEV-NatMX pEM988(SUC2) | This study |
| JKS129 | | MATa ura3-52 lys2-801am ade2-101oc trp1Δ1 his-A200 sec63Δ::KANXM SEC63wt-pEM837(Ura) SEC63-Δ142-pRS315(LEU) gal1Δ::TEV-HphMX4 leu2Δ0::PACT1-GEV-NatMX | This study |
| JKS128 | | MATa ura3-52 lys2-801am ade2-101oc trp1Δ1 his-A200 sec63Δ::KANXM SEC63wt-pEM837(Ura) SEC63-Δ142-HA-pRS315(LEU) gal1Δ::TEV-HphMX4 leu2Δ0::PACT1-GEV-NatMX | This study |
| JKS134 | | MATa ura3-52 lys2-801am ade2-101oc trp1Δ1 his-A200 sec63Δ::KANXM SEC63wt-pEM837(Ura) SEC63-Δ142-GFP-pRS315(LEU) gal1Δ::TEV-HphMX4 leu2Δ0::PACT1-GEV-NatMX | This study |
| JKS117 | | MATa ura3-52 lys2-801am ade2-101oc trp1Δ1 his-A200 sec63Δ::KANXM SEC63wt-pEM837(Ura) SEC63-Δ202-pRS315(LEU) gal1Δ::TEV-HphMX4 leu2Δ0::PACT1-GEV-NatMX | This study |
| JKS111 | | MATa ura3-52 lys2-801am ade2-101oc trp1Δ1 his-A200 sec63Δ::KANXM SEC63wt-pEM837(Ura) SEC63-Δ202 pRS315(LEU) gal1Δ::TEV-HphMX4 leu2Δ0::PACT1-GEV-NatMX pEM778(DPAPB) | This study |
| JKS112 | | MATa ura3-52 lys2-801am ade2-101oc trp1Δ1 his-A200 sec63Δ::KANXM SEC63wt-pEM837(Ura) SEC63-Δ202-pRS315(LEU) gal1Δ::TEV-HphMX4 leu2Δ0::PACT1-GEV-NatMX pEM952(GAS1) | This study |
| JKS113 | | MATa ura3-52 lys2-801am ade2-101oc trp1Δ1 his-A200 sec63Δ::KANXM SEC63wt-pEM837(Ura) SEC63-Δ202 pRS315(LEU) gal1Δ::TEV-HphMX4 leu2Δ0::PACT1-GEV-NatMX pEM953(PHO8) | This study |
| JKS114 | | MATa ura3-52 lys2-801am ade2-101oc trp1Δ1 his-A200 sec63Δ::KANXM SEC63wt-pEM837(Ura) 6'-SEC63-pRS315(LEU) gal1Δ::TEV-HphMX4 leu2Δ0::PACT1-GEV-NatMX pEM988 (SUC2) | This study |
| JKS140 | | MATa ura3-52 lys2-801am ade2-101oc trp1Δ1 his-A200 sec63Δ::KANXM SEC63wt-pEM837(Ura) SEC63-Δ237-GFP-pRS315(LEU) gal1Δ::TEV-HphMX4 leu2Δ0::PACT1-GEV-NatMX | This study |
| JKS141 | | MATa ura3-52 lys2-801am ade2-101oc trp1Δ1 his-A200 sec63Δ::KANXM SEC63wt-pEM837(Ura) SEC63-Δ237-GFP-pRS315(LEU) gal1Δ::TEV-HphMX4 leu2Δ0::PACT1-GEV-NatMX pEM778 (DPAPB) | This study |
| JKS142 | | MATa ura3-52 lys2-801am ade2-101oc trp1Δ1 his-A200 sec63Δ::KANXM SEC63wt-pEM837(Ura) SEC63-Δ237-GFP-pRS315(LEU) gal1Δ::TEV-HphMX4 leu2Δ0::PACT1-GEV-NatMX pEM952 (GAS1) | This study |
| JKS143 | | MATa ura3-52 lys2-801am ade2-101oc trp1Δ1 his-A200 sec63Δ::KANXM SEC63wt-pEM837(Ura) SEC63-Δ237-GFP-pRS315(LEU) gal1Δ::TEV-HphMX4 leu2Δ0::PACT1-GEV-NatMX pEM953 (PHO8) | This study |
| JKS144 | | MATa ura3-52 lys2-801am ade2-101oc trp1Δ1 his-A200 sec63Δ::KANXM SEC63wt-pEM837(Ura) SEC63-Δ237-GFP-pRS315(LEU) gal1Δ::TEV-HphMX4 leu2Δ0::PACT1-GEV-NatMX pEM988(SUC2) | This study |
