# Supplementary material for: Rapid inactivation of the yeast Sec complex selectively blocks transport of post-translationally translocated proteins
Source: J Biol Chem. 2021 Sep 4;297(4):101171. doi: 10.1016/j.jbc.2021.101171 (PMC8503631; doi:10.1016/j.jbc.2021.101171)
Supplement: Supplemental Table S2 [file mmc2.docx]

**TableS2**. Primers used in this study.

| PrimerID | Sequence |
| --- | --- |
| sGAL1-F | AAAGAGCCCCATTATCTTAGCCT |
| sGAL1-R | TCCCTGTGTTTTAAAGTTTGTGGA |
| sTEV-F | GGCAGTGTGGCAGTCCATTA |
| sTEV-R | CACTGCCCATCCTTGGTTTG |
| tCYCt-F | TAGTTATGTCACGCTTACATTCACGC |
| tCYCt-R | CCTAGACTTCAGGTTGTCTAACTCC |
| tDownstream-GAL1 | CATTTCTTTTCCTCCTCGCGC |
| tUpstream-GAL1 | GTATTGCAGGGCTGCAATATGAC |
| ymUKG-F-Phospho | /5Phos/ATGGTCAGTGTCATCAAAGAAGA |
| ymUKG-R-Phospho | /5Phos/CTTAGAAGCTTGAGATGGCAAC |
| Sec63-Δ35(SGSG-TEVs-SGSG)-F | TCTGAGGAGGATGATGAGAGCGGCGAAAACCTGTACTTTCAGGGCGGCAGCTACTCTACTGATGACGAC |
| Sec63-Δ35(SGSG-TEVs-SGSG)-R | GTCGTCATCAGTAGAGTAGCTGCCGCCCTGAAAGTACAGGTTTTCGCCGCTCTCATCATCCTCCTCAGA |
| Sec63-Δ52(SGSG-TEVs-SGSG)-F | ATGAATATGAAAGTTCGTAGCGGCGAAAACCTGTACTTTCAGGGCGGCAGCGATTCTCC |
| Sec63-Δ52(SGSG-TEVs-SGSG)-R | TTCCACTGCAGGAGAATCGCTGCCGCCCTGAAAGTACAGGTTTTCGCCGCTACGAACTTTCATATTCAT |
| Sec63-Δ142(GS-TEVs-GS-SF3b)-F | CTTTCTGATGAGGAATTAGATGCTATGTTCCCAGAAGGATATAAGGTACTTCCTCCTCCAGCTGGTTATGTTCCTATTCGAACTCCAGCTGGTTCTGATGGTAAAATACTTCAAACGCCAATTATCATTGAAAAGC |
| Sec63-Δ142(GS-TEVs-GS-SF3b)-R | TGGGCGATTTCTCTCATCAATTTCTCTTTCCCACCGCCAAGCCTGAAGCTGTTCTTTATAATCAGACTTGTGGCCCTGAAAGTACAGGTTTTCAGAACCTTTTTGAGAACTTACCAGACAGCACCAACTC |
| Sec63-Δ202(GS-TEVs-GS-SF3b)-F | CTTTCTGATGAGGAATTAGATGCTATGTTCCCAGAAGGATATAAGGTACTTCCTCCTCCAGCTGGTTATGTTCCTATTCGAACTCCAGCTGGTTCTCAGCCATTGATACCAACTAGCTTAATTCC |
| Sec63-Δ202(GS-TEVs-GS-SF3b)-R | TGGGCGATTTCTCTCATCAATTTCTCTTTCCCACCGCCAAGCCTGAAGCTGTTCTTTATAATCAGACTTGTGGCCCTGAAAGTACAGGTTTTCAGAACCTTTAGCAGAACGAACCAGTACTTTCAAAGAAATG |
| Sec63-Δ237(GS-TEVs-GS-SF3b)-F | CTTTCTGATGAGGAATTAGATGCTATGTTCCCAGAAGGATATAAGGTACTTCCTCCTCCAGCTGGTTATGTTCCTATTCGAACTCCAGCTGGTTCTAATCTAAAGATCATCAAGGCAGACTTCCTTGTC |
| Sec63-Δ237(GS-TEVs-GS-SF3b)-R | TGGGCGATTTCTCTCATCAATTTCTCTTTCCCACCGCCAAGCCTGAAGCTGTTCTTTATAATCAGACTTGTGGCCCTGAAAGTACAGGTTTTCAGAACCTGGAATATGCGATGCAACTCTCAAAGTTTCG |
| Sec63-RE-R | TTCTGGTGATTCATCATC |
| Sec63-RE-blunt-F | TAGATATAAGTGATTAGTATAGTTTTTC |
| Sec63-Δ237-short1F | CTTGTGGCCCTGAAAGTACAGGTTTTC |
| Sec63-Δ237-short1R | CGCCCACTTTCTGATGAGGAATTAGATG |
| Sec63-Δ237-short2F | AAGTACCTTATATCCTTCTGGGAACATAGCATCTAATTC |
| Sec63-Δ237-short2R | AATCTAAAGATCATCAAGGCAGACTTCCTTGTC |
| Sec63-3XHA-F | CCGGACTATGCAGGATCCTATCCATATGACGTTCCAGATTACGCTTAGATATAAGTGATTAGTATAGTTTTTC |
| Sec63-3XHA-R | GACGTCATACGGATAGCCCGCATAGTCAGGAACATCGTATGGGTATTCTGGTGATTCATCATC |
